# Morphology, morphogenesis, and multigene phylogeny of *Stichotricha koreana* sp. nov. (Alveolata, Ciliophora, Hypotricha): taxonomic implications for the family Chaetospiridae Jankowski in Small & Lynn, 1985

**DOI:** 10.3897/zookeys.1275.181694

**Published:** 2026-04-02

**Authors:** Daizy Bharti, Santosh Kumar, Atef Omar, Jae-Ho Jung

**Affiliations:** 1 Zoological Survey of India, Prani Vigyan Bhawan, M-Block, New Alipore, Kolkata 700053, India Zoological Survey of India Kolkata India; 2 Department of Biological Sciences, Kangwon National University, Gangneung 25457, Republic of Korea Department of Biological Sciences, Kangwon National University Gangneung Republic of Korea; 3 EcoBio Research Center, Kangwon National University, Gangneung 25457, Republic of Korea EcoBio Research Center, Kangwon National University Gangneung Republic of Korea

**Keywords:** Gonostomatid oral apparatus, integrative taxonomy, loricate hypotrichs, semi-terrestrial ciliate, South Korea

## Abstract

The morphology, morphogenesis, and multi-gene phylogeny of a new hypotrich, *Stichotricha koreana***sp. nov**., from a temporary pond in Gangneung-si, South Korea, were investigated. The new species inhabits a gelatinous lorica that is often branched, forming a colony. Diagnostic features include two frontal cirri, one buccal cirrus, a short parabuccal row, two frontoventral cirral rows, a single marginal row on each side, and three dorsal kineties, each with a caudal cirrus. The frontoventral rows, marginal rows, and dorsal kineties spiral by half to one turn around the long body axis. Detailed morphogenesis is documented in the genus for the first time: the oral primordium arises close to the left frontoventral row; the parental adoral zone is fully retained; frontoventral rows, both marginal rows, and all dorsal kineties develop at two levels via within-row anlagen formation; and the parabuccal row originates from the oral primordium. Phylogenetic analyses of 18S rDNA place *Stichotricha koreana***sp. nov**. within Chaetospiridae, forming a sister group to members of the family Gonostomatidae and the core urostylids. A concatenated rDNA gene dataset shows that Chaetospiridae is sister to hypotrichs with gonostomatid oral apparatus. These data refine the diagnosis of Chaetospiridae and confirm the generic assignment of *S. koreana***sp. nov**.

## Introduction

The placement of *Stichotricha* and related taxa among the hypotrich ciliates has been repeatedly revised, largely because many species lacked modern documentation based on live observation, silver impregnation, and molecular data (Bourland 2015; Song et al. 2022). Jankowski (1979) established the Stichotrichinae as a subfamily of Strongylidiidae Fauré-Fremiet, 1961 with *Stichotricha* Perty, 1849 as the name-bearing type genus, but later abandoned this concept (Jankowski 2007). Borror and Evans (1979) informally proposed to separate the “top-shaped” spirofilids (i.e., *Hypotrichidium* and *Microspiretta*) from the slender, tubicolous lineages (i.e., *Stichotricha* and *Chaetospira* Lachmann, 1856) into different families, although they did not formalize this as a nomenclatural act. Subsequently, Lynn and Small (2002) and Lynn (2008) accepted the classification of Corliss (1960) in placing *Stichotricha* in Spirofilidae Gelei, 1929. However, Bourland (2015) documented that *Stichotricha aculeata* Wrześniowski, 1866 is morphologically distinct from typical spirofilids, although the information available to Bourland (2015) was insufficient to assign it confidently to an established family or to justify the erection of a new one. More recent integrative work has clarified part of this taxonomic framework by resurrecting the family Chaetospiridae Jankowski in Small & Lynn, 1985 and transferring *Chaetospira* and *Stichotricha* to it (Song et al. 2022).

Since the establishment of *Stichotricha* by Perty (1849), eleven nominal species have been assigned to the genus, most of which are poorly documented except for *S. aculeata* Wrześniowski, 1866. The species currently attributed to *Stichotricha* are as follows: (1) *S. aculeata*; (2) *S. asensius* Fernandez-Leborans, 1985; (3) *S. gracilis* Möbius, 1888; (4) *S. intermedia* Froud, 1949; (5) *S. marina* Stein, 1867; (6) *S. multinucleata* Song & Wilbert, 1989; (7) *S. nankingensis* Wang & Nie, 1933; (8) *S. opisthotonoides* Smith, 1897; (9) *S. saginata* Möbius, 1888; (10) *S. secunda* Perty, 1849 (type); and (11) *S. tubicola* (Gruber, 1880) Borror, 1972 (Perty 1849; Entz 1884; Froud 1949; Borror 1972; Berger 1999, 2001; Hu and Song 2001). However, the 18S rDNA sequence of the type species, *S. secunda*, remains unavailable, and the generic placement of these taxa requires confirmation through integrative revisions that combine detailed live and silver-impregnation-based morphology, morphogenetic data, and additional molecular markers.

Here, we describe a new species of *Stichotricha* discovered in a temporary pond in South Korea. Using an integrative approach, we provide a comprehensive morphological characterization based on live observations, protargol preparations, and scanning electron microscopy (SEM). From silver-impregnated material, we also documented, for the first time in the genus, the sequence of ontogenetic events. Finally, phylogenetic analyses of the 18S rDNA, ITS1–5.8S–ITS2 region, 18S–ITS1–5.8S–ITS2 rDNA, 28S rDNA, and a concatenated nuclear rDNA dataset (18S–ITS1–5.8S–ITS2–28S) generated for the new species and a Korean population of *Stichotricha aculeata*, collected from the same locality where the population reported by Omar and Jung (2021) was found on 19 August 2019, corroborate the placement of *Stichotricha* within Chaetospiridae, as proposed by Song et al. (2022), and reveal a close relationship to hypotrich families possessing a gonostomatid oral apparatus.

## Materials and methods

### Sample collection, cultivation, preparation, and identification

*Stichotricha koreana* sp. nov. was discovered in a water sample collected from a temporary puddle on a footpath (Baugil) behind the Kangwon National University, Gangneung-si, South Korea, on 21 July 2022. The sample was immediately transported to the laboratory and raw cultures were established in Plant Culture Dishes (SPL Life Sciences, Gyeonggi-do, Korea; 100 mm diameter × 40 mm depth) at room temperature. A culture containing few cells of the new species, *Cyclidium* sp., and flagellates, was established in a Plant Culture Dish, using mineral water (Jeju Samdasoo, Jeju Province Development Co., South Korea), and enriched with sterilized squashed wheat kernels and mealworms. Living specimens were examined using a stereomicroscope (Olympus SZ61, Tokyo, Japan) and a light microscope (Olympus BX53) with differential interference contrast (DIC) at magnifications of 50–1,000×. The infraciliature was revealed through protargol impregnation and SEM. Protargol powder was synthesized following the methods of Kim and Jung (2017) and Pan et al. (2013), and the impregnation was performed using ‘Procedure A’ from Foissner (2014). SEM was conducted according to Foissner (2014) and Moon et al. (2020).

Terminology follows Foissner and Al-Rasheid (2006) and Lynn (2008). Classification adheres to Lynn (2008) and Song et al. (2022). The new species has only two frontal cirri and lacks the third (right) frontal cirrus that typically originates from anlage III in most other hypotrichs. Therefore, we designate these as frontal cirrus 1 (originating from anlage I) and frontal cirrus 2 (originating from anlage II).

### DNA extraction, PCR amplification, and sequencing

Five cells of *Stichotricha koreana* sp. nov. were isolated from the culture, and five cells from a population of *S. aculeata*, collected from the same locality as the population reported by Omar and Jung (2021), were obtained from the environmental sample using glass micropipettes under a stereomicroscope. The cells were washed at least five times with sterile distilled water and then each cell was transferred to a 1.5 mL centrifuge tube with a minimum volume of water. Genomic DNA was extracted using a REDExtract-N-Amp Tissue PCR Kit (Sigma, St. Louis, MO, USA). The 18S rDNA was amplified using the New Euk A forward primer (5'-CTG GTT GAT YCT GCC AGT-3') (Jung and Min 2009) and the LSU rev4 reverse primer (5'-GTT AGA CTY CTT GGT CCG TG-3') (Sonnenberg et al. 2007), covering nearly the entire 18S–ITS1–5.8S–ITS2 rDNA. The PCR conditions followed the study of Omar et al. (2025). The 28S rDNA was amplified using the primers 28S-1F and 28S-4R (Moreira et al. 2007). The PCR conditions were as follows: denaturation at 94 °C for 5 min, followed by 40 cycles of denaturation at 94 °C for 15 s, annealing at 58 °C for 1 min, and extension at 72 °C for 2 min, and a final extension step at 72 °C for 7 min. The PCR products were purified using the MEGAquick-spin Total Fragment DNA Purification Kit (iNtRON, South Korea). Since the resulting sequence fragments obtained using the New Euk A were identical among the five cells of each species, sequencing of other fragments was performed bidirectionally on the same cell for each species on an ABI 3700 sequencer (Applied Biosystems, Foster City, CA, USA). For the first primer set, internal sequencing primers 18SR300, 18SF790v2, and 18SF1470 were used (Park et al. 2017; Jung et al. 2018). For the second primer set, internal primers 28S-568F and 28S-2F were used. Resulting sequence fragments from the two sets were assembled using Geneious ver. 9.1.8 (Kearse et al. 2012).

### Phylogenetic analyses

To determine the phylogenetic position of the two species, five datasets were prepared. The first 18S rDNA dataset consists of 136 sequences (1711 characters). The other four datasets each comprised 63 taxa: (ii) ITS1–5.8S rDNA–ITS2 (661 characters), (iii) 18S–ITS1–5.8S–ITS2 rDNA (2424 characters), (iv) 28S rDNA (1850 characters), and (v) a concatenated nuclear rDNA dataset (18S–ITS1–5.8S–ITS2–28S) sequences taxa (4288 characters). Three euplotids were used as outgroup taxa in each dataset. The sequences were aligned using ClustalW (Thompson et al. 1994), and both ends were manually trimmed in BioEdit ver. 7.0.9.0 (Hall 1999). jModelTest ver. 2.1.7 (Darriba et al. 2012) was used to select the best-fit model, GTR + I + G for all datasets except that of 28S rDNA, for which the GTR + G model is selected, under the Akaike information criterion (AIC). The maximum likelihood (ML) trees of both datasets were constructed using IQ-Tree ver. 1.6.11 (Nguyen et al. 2015) with 100,000 ultrafast bootstrap replicates. The pairwise sequence similarity among taxa was calculated using MEGA ver. 6.06 (Tamura et al. 2013). Bayesian inference (BI) analyses were performed using MrBayes ver. 3.1.2 (Ronquist et al. 2012) with Markov chain Monte Carlo simulations running for 3,000,000 generations, sampling every 100 generations, with the first 7,500 trees discarded as burn-in. Phylogenetic trees were visualized with MEGA ver. 6.06 and the free software package FigTree ver. 1.4.3 (A. Rambaut at http://tree.bio.ed.ac.uk/software/figtree/). Interpretation of bootstrap values and Bayesian posterior probabilities followed Hillis and Bull (1993) and Alfaro et al. (2003), respectively.

## Results

### Taxonomy

**Phylum Ciliophora Doflein, 1901**

**Class Spirotrichea Bütschli, 1889**

**Subclass Hypotrichia Stein, 1859**

**Order Stichotrichida Fauré-Fremiet, 1961**

#### Family Chaetospiridae

Taxon classificationAnimaliaStichotrichidaChaetospiridae

Jankowski in Small & Lynn, 1985

D7BDA5C5-ABA7-5134-9FE2-C885DEFA1A02

##### Improved diagnosis.

Non-dorsomarginalian Hypotrichia with flask-shaped and distinctly spiraled body. Lorica usually present. Oral apparatus gonostomatid, i.e., with adoral zone extending along left margin of narrow neck-like region, paroral and endoral stichomonad, paroral basal bodies more widely spaced than those of endoral. Frontal and parabuccal cirri present or absent. Two frontoventral and two marginal cirral rows. Pretransverse and transverse cirri absent. Caudal cirri present or absent. Frontoventral, marginal, and dorsal kinety rows originate at two levels through ‘within-row’ anlagen formation.

##### Genera included.

*Chaetospira* Lachmann, 1856 and *Stichotricha* Perty, 1849.

### Genus *Stichotricha* Perty, 1849

#### Stichotricha koreana sp. nov.

Taxon classificationAnimaliaStichotrichidaSpirofilidae

4FC9BDE6-44CB-5D6A-935D-2999741BD407

https://zoobank.org/BBC357E1-A27F-4EF5-936B-2B844E48A4E6

Figs 1, 2, 3, 4, 5, 6, 7, 8, 9; Table 1

##### Diagnosis.

Body size 172–217 × 42–62 µm in vivo. Body slender and elongate. Cell inhabiting a cylindrical, gelatinous lorica. Two macronuclear nodules and two to five micronuclei. Adoral zone occupying ~ 58% of body length, composed of 71–94 membranelles. Endoral ~ 30% shorter than paroral anteriorly. Two frontal and one or two buccal cirri. Parabuccal row in narrowed anterior region, composed of 6–12 cirri. Left frontoventral row composed of 57–74 and right row of 55–75 cirri. Left marginal cirral row composed of 46–57, right row of 43–59 cirri. Three bipolar dorsal kineties, each with one caudal cirrus posteriorly.

##### Type locality.

Temporary puddle (after rainfall) on a footpath (Baugil) behind the Kangwon National University, Gangneung-si, Korea (37°46'27.80"N, 128°51'54.00"E).

##### Type material.

**Figure 1.**
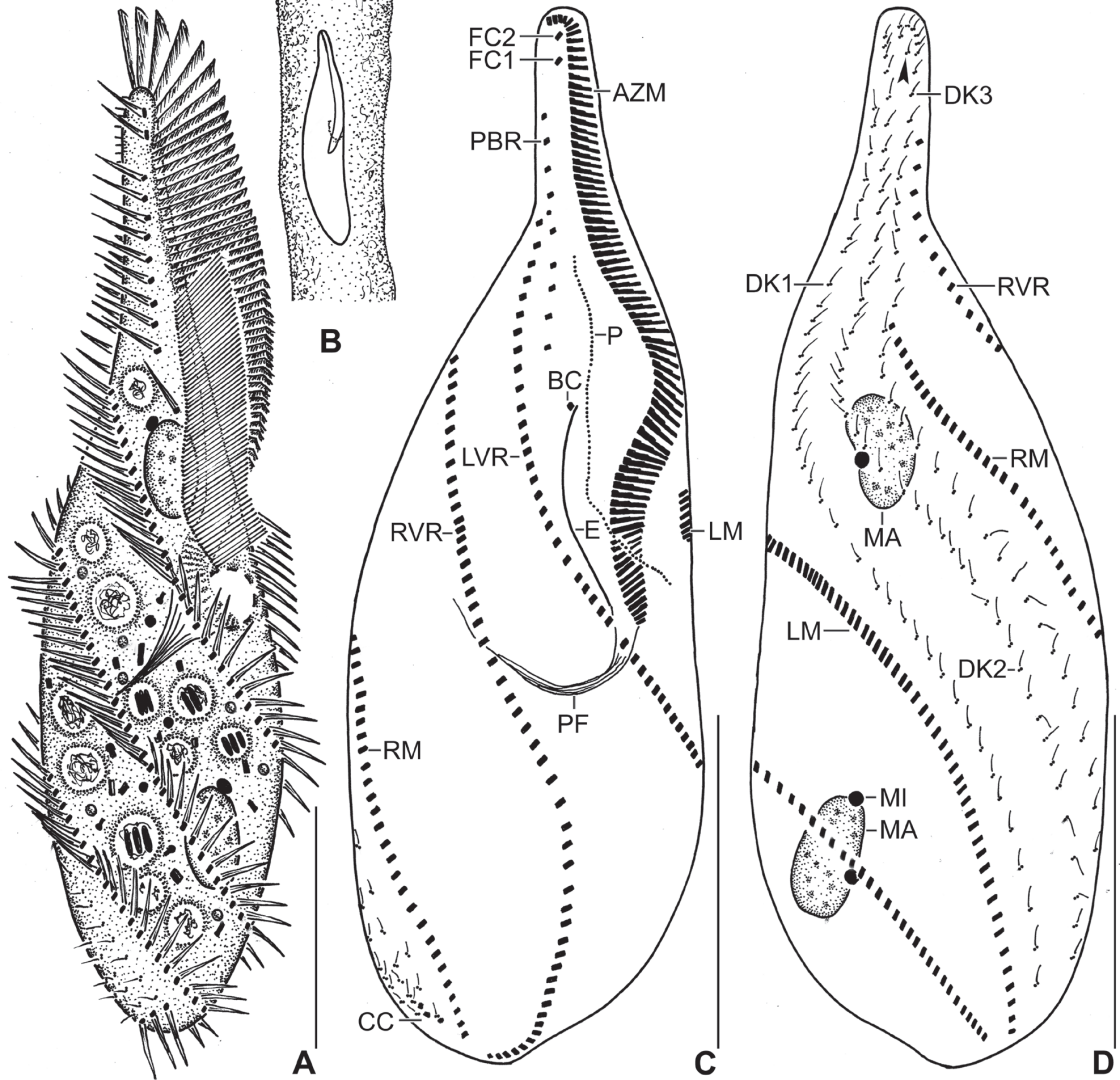
Line drawings of *Stichotricha koreana* sp. nov. from life (**A, B**) and after protargol impregnation (**C, D**). **A**. Ventral view of a representative specimen, length 194 µm; **B**. Specimen within lorica; **C, D**. Ventral (**C**) and dorsal (**D**) view of the holotype specimen. Note the long narrow anterior portion of body, monokinetid paroral membrane, ciliary rows making ~ half to one body turn, and three bipolar dorsal kineties. The arrowhead in (**D**) indicates the presence of 2–4 bristles between kineties 2 and 3. Abbreviations: AZM, adoral zone of membranelles; BC, buccal cirrus; CC, caudal cirri; DK1–3, dorsal kineties 1–3; E, endoral membrane; FC1, 2, frontal cirri; LVR, left frontoventral row; LM, left marginal row; MA, macronuclear nodules; MI, micronuclei; P, paroral membrane; PBR, parabuccal cirral row; PF, pharyngeal fibers; RM, right marginal row; RVR, right frontoventral row. Scale bars: 50 µm.

The protargol slide (NNIBRPR27999) with the holotype specimen (Figs 1C, 1D, 3A, 3B) and two paratype slides (NNIBRPR28000, NNIBRPR28001) have been deposited at the Nakdonggang National Institute of Biological Resources, Korea. The holotype and paratype specimens have been marked by black ink circles on the back of the slides. Additionally, five other paratype slides (GUC007031, GUC007032, GUC007035, GUC007047, and GUC007048) have been deposited in Jung lab (J.-H. Jung) at the Kangwon National University.

##### Etymology.

The species-group name *koreana* refers to the country where the species was discovered.

##### Description.

**Figure 2.**
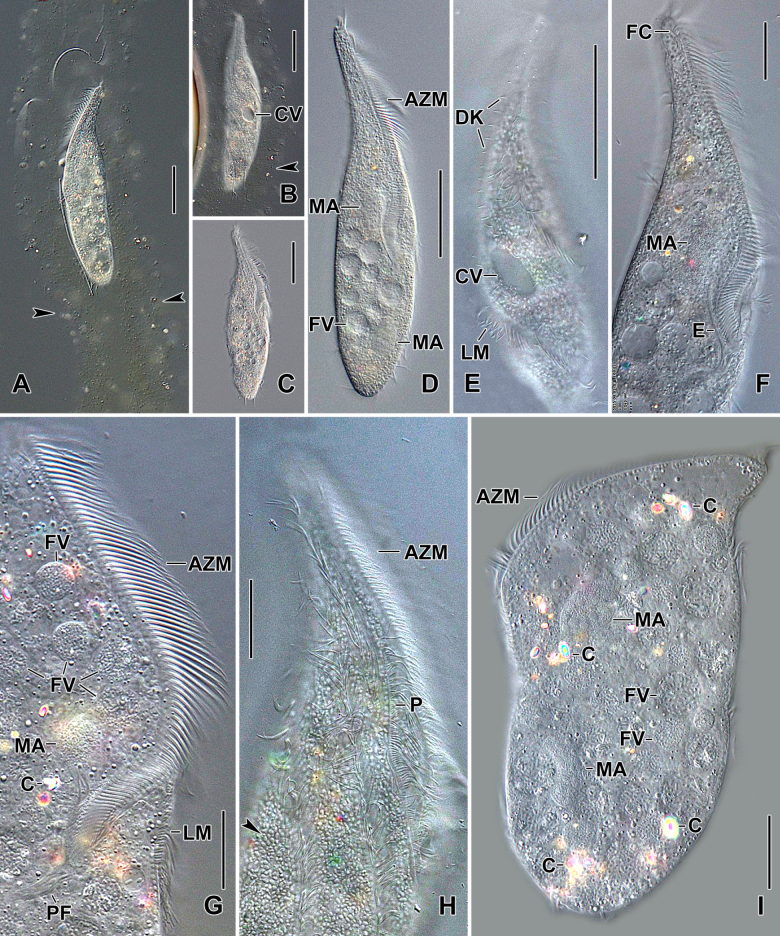
Photomicrographs of *Stichotricha koreana* sp. nov. from life under the DIC illumination. **A, B**. Specimens in tube-like lorica; arrowheads mark lorica margin; **C, D**. Ventral views of specimens showing body shape; **E**. Dorsal view showing the contractile vacuole and dorsal bristles; **F, G**. Ventral views showing the adoral zone of membranelles similar to that of the gonostomatids, i.e., straight extending along narrow portion of body with proximal end curved toward mid-body; **H**. Ventral view showing details of paroral membrane and ventral ciliary rows; arrowhead points to sub-pellicular mitochondria (?) covering entire cell; **I**. Dorsal view of a specimen pressed by coverslip, revealing cytoplasmic crystals, macronuclear nodules, and numerous food vacuoles. Abbreviations: AZM, adoral zone of membranelles; C, crystals; CV, contractile vacuole; DK, dorsal kineties; E, endoral membrane; FC, frontal cirrus; FV, food vacuole; LM, left marginal row; MA, macronuclear nodules; P, paroral membrane; PF, pharyngeal fibers. Scale bars: 50 µm (**A–E, I**) and 20 µm (**F–H**).

**Figure 3.**
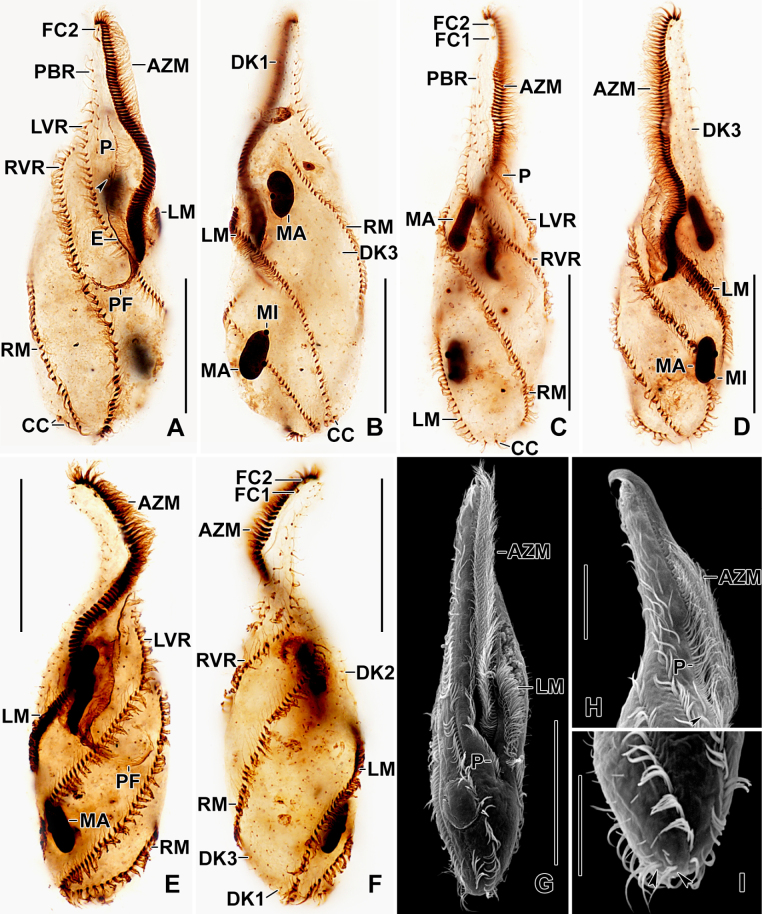
Photomicrograph of *Stichotricha koreana* sp. nov. after protargol impregnation (**A–F**) and scanning electron microscopy (**G–I**). **A–F**. Ventral and dorsal views of the holotype (**A, B**) and paratype specimens (**C–F**) showing ciliature and nuclear apparatus, long narrow anterior body portion, and the distal portion of adoral zone of membranelles containing three or four membranelles is curved rightwards. Arrowhead in (**A**) points to buccal cirrus; **G**. Ventral view showing details of body shape, ciliature, and buccal cavity; **H**. Ventral view of the narrow region, showing the adoral zone and cirri in frontal region; arrowhead points to buccal cirrus; **I**. Dorsolateral view of posterior portion, showing dorsal kineties and caudal cirri (arrowheads). Abbreviations: AZM, Adoral zone of membranelles; CC, caudal cirrus; DK1-3, dorsal kineties 1-3; E, endoral membrane; FC1, 2, frontal cirri; LVR, left frontoventral row; LM, left marginal row; MA, macronuclear nodules; MI, micronuclei; P, paroral membrane; PBR, parabuccal cirral row; PF, pharyngeal fibers; RM, right marginal row; RVR, right frontoventral row. Scale bars: 50 µm (**A–F**) and 20 µm (**H, I**).

**Figure 4.**
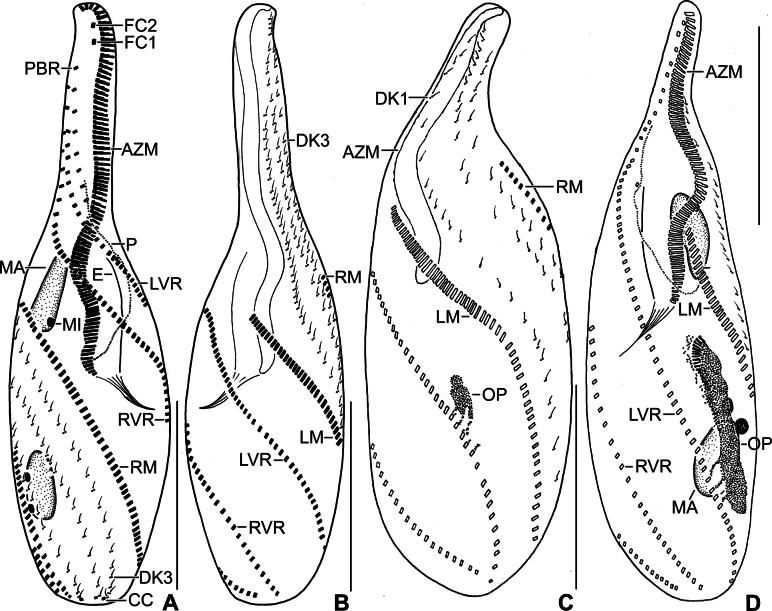
Line drawings of a protargol-impregnated morphostatic specimen (**A, B**) and dividers (**C, D**) of *Stichotricha koreana* sp. nov. **A, B**. Ventral (**A**) and dorsal (**B**) view showing body shape and ciliature; **C**. Ventral view of very early divider; note formation of oral primordium near left frontoventral row with contribution from 4–7 cirri; **D**. Ventral view of an early divider, showing the line of disoriented basal bodies right to the anterior half of oral primordium and the formation of new adoral membranelles at anterior end of oral primordium. Abbreviations: AZM, adoral zone of membranelles; CC, caudal cirrus; DK1, 3, dorsal kineties 1.3; E, endoral membrane; FC1, 2, frontal cirri; LVR, left frontoventral row; LM, left marginal row; MA, macronuclear nodules; MI, micronuclei; OP, oral primordium; P, paroral membrane; PBR, parabuccal cirral row; RM, right marginal row; RVR, right frontoventral row. Scale bars: 50 µm.

Body size in vivo 172–217 × 42–62 µm (on average 194 × 52 µm) and 136–170 × 36–55 µm after protargol impregnation. Length:width ratio 3.0–4.5:1 in vivo (on average 3.8:1) and 2.9–4.1:1 (on average 3.4:1) after protargol impregnation (Figs 1A, 1C, 1D, 2A–D, 3A–G, 4A, 4B; Table 1). Body slender to elongate fusiform, mid-body circular in cross section. Anterior body portion narrow and slightly dorsally curved bearing more than half of the adoral zone of membranelles (AZM); mid-body with slightly to distinctly convex margins; posterior end rounded (Figs 1A, 1C, 1D, 2A–H, 3A–H, 4A, 4B). Cell flexible and slightly contractile (Figs 2A–H). Nuclear apparatus consisting of two widely separated macronuclear nodules and two to five micronuclei, located in the second and fourth quarters of the body, slightly left of mid-line. Macronuclear nodules ellipsoidal, ~ 18 × 9 µm in protargol preparations, each containing numerous nucleoli. Micronuclei spherical, usually attached to macronuclear nodules ~ 2.3 × 2.1 µm in protargol preparations (Figs 1A, 1D, 2D, 2F, 2G, 2I, 3B–F, 4A; Table 1). Contractile vacuole near mid-body at left body margin; collecting canals extending to near both body ends (Figs 1A, 2B, 2E). Cortical granules absent; numerous subpellicular mitochondria present (Fig. 2H). Cytoplasm colorless, containing small refractive crystals and few fat droplets (Figs 1A, 2G, 2I). Food vacuoles containing bacteria (Figs 1A, 2D, 2G, 2I). Cells inhabit yellowish, granular, translucent gelatinous loricae, 100–140 µm wide in vivo, often branched (Figs 1B, 2A, 2B). Cells move forward and backward within the lorica, often escaping by swimming moderately fast while rotating around the longitudinal axis. Multiple loricae usually attach together forming a colony and occasionally connect at their bases.

Adoral zone straight, distal end slightly extending to right, occupying ~ 58% of body length, composed of 71–94 membranelles. Cilia ~ 14 µm long in vivo. Buccal cavity deep, tear-drop shaped at base of narrowed anterior portion of body. Undulating membranes each composed of a single row of basal bodies, basal bodies in the paroral spaced more widely than those in the endoral; paroral 43–56 µm long, cilia 12–14 µm long in vivo; endoral 29–39 µm long, slightly sigmoid, shortened anteriorly (Figs 1A, 1C, 2D, 2F–H, 3A, 3D, 3E, 3G, 3H, 4A; Table 1). Pharyngeal fibers curved towards right (Figs 1A, 2F, 2G, 3A, 3E).

**Table 1.** Morphometric data on *Stichotricha koreana* sp. nov.

Characteristic^a^	HT	Mean	M	SD	SE	CV	Min	Max	*n*
Body, length (in vivo)	-	193.5	196.0	14.5	4.4	7.5	172.0	217.0	11
Body, width (in vivo)	-	51.9	52.0	8.5	2.6	16.3	42.0	62.0	11
Body length:width, ratio (in vivo)	-	3.8	3.8	0.5	0.2	13.7	3.0	4.5	11
Body, length	159.0	153.0	154.0	11.3	2.4	7.4	136.0	170.0	23
Body, width	55.0	45.3	46.0	4.9	1.0	10.7	36.0	55.0	23
Body length:width, ratio	2.9	3.4	3.4	0.3	0.1	9.4	2.9	4.1	23
Anterior body end to proximal end of AZM, distance	94.0	88.0	86.0	7.7	1.6	8.7	78.0	110.0	23
Anterior body end to proximal end of AZM, % of body length	59.1	57.7	56.9	4.3	0.9	7.4	50.0	65.1	23
Adoral membranelles, width of largest base	5.5	5.5	5.5	0.6	0.1	10.9	4.5	7.0	21
Adoral membranelles, number	79.0	79.4	79.0	4.9	1.1	6.2	71.0	94.0	21
Paroral membrane, length	52.0	47.7	47.0	3.6	0.8	7.5	43.0	56.0	19
Anterior body end to PM, distance	37.0	35.6	35.0	5.2	1.2	14.6	28.0	48.0	19
Endoral membrane, length	36.0	32.6	32.0	2.5	0.5	7.6	29.0	39.0	21
Anterior body end to EM, distance	58.0	55.0	54.0	5.3	1.1	9.6	48.0	70.0	21
Nuclear figure, length	80.0	72.0	70.0	10.7	2.3	14.9	48.0	95.0	21
Anterior body end to anterior macronuclear nodule, distance	57.0	57.4	56.0	6.0	1.3	10.4	50.0	74.0	21
Posterior body end to posterior macronuclear nodule, distance	25.0	21.5	21.0	3.3	0.7	15.2	15.0	29.0	21
Anterior macronuclear nodule, length	18.0	17.7	17.0	2.7	0.6	15.4	12.0	23.0	19
Anterior macronuclear nodule, width	10.0	8.7	9.0	1.4	0.3	16.3	7.0	12.0	19
Posterior macronuclear nodule, length	20.0	17.7	18.0	1.9	0.4	10.9	14.0	21.0	19
Posterior macronuclear nodule, width	10.0	8.8	9.0	1.5	0.4	17.6	6.0	11.0	19
Macronuclear nodules, number	2.0	2.0	2.0	0.0	0.0	0.0	2.0	2.0	21
Anteriormost micronucleus, length	2.5	2.3	2.2	0.2	0.0	7.7	2.0	2.5	19
Anteriormost micronucleus, width	2.0	2.1	2.0	0.1	0.0	6.3	2.0	2.5	19
Micronuclei, number	3.0	3.6	3.0	0.9	0.2	25.9	2.0	5.0	21
Anterior body end to right marginal row, distance	47.0	54.6	54.0	7.1	1.6	13.1	44.0	71.0	21
Right marginal row, number of cirri	56.0	53.0	54.0	4.0	0.9	7.6	43.0	59.0	21
Anterior body end to left marginal row, distance	74.0	68.2	66.0	8.8	1.9	12.9	55.0	90.0	21
Left marginal row, number of cirri	54.0	52.5	54.0	3.5	0.8	6.8	46.0	57.0	21
Frontal cirri, number	2.0	2.0	2.0	0.0	0.0	0.0	2.0	2.0	21
Anterior body end to anterior BC, distance	60.0	55.8	55.0	5.1	1.1	9.1	48.0	70.0	21
Anterior end of EM to anterior BC, distance	2.0	0.9	1.0	0.9	0.2	99.6	0.0	3.0	21
Buccal cirri, number	1.0	1.1	1.0	0.3	0.1	28.5	1.0	2.0	19
Anterior body end to parabuccal row, distance	15.0	16.1	15.0	5.0	1.2	31.2	10.0	31.0	19
Parabuccal row, number of cirri	10.0	8.9	9.0	1.4	0.3	15.4	6.0	12.0	19
Frontoventral rows, number	2.0	2.0	3.0	0.0	0.0	0.0	2.0	2.0	21
Anterior body end to left frontoventral row, distance	31.0	30.0	31.0	6.7	1.5	22.3	18.0	39.0	19
Left frontoventral row, number of cirri	68.0	65.5	66.0	4.2	1.0	6.4	57.0	74.0	19
Anterior body end to right frontoventral row, distance	17.0	20.6	21.0	5.1	1.2	24.5	12.0	30.0	17
Right frontoventral row, number of cirri	65.0	63.5	64.0	5.5	1.3	8.7	55.0	75.0	19
Dorsal kineties, number	3.0	3.0	3.0	0.0	0.0	0.0	3.0	3.0	21
Anterior body end to dorsal kinety 1, distance	15.0	19.1	18.0	5.3	1.3	27.8	11.0	29.0	17
Dorsal kinety 1, number of bristles	41.0	41.6	41.0	3.2	0.8	7.7	38.0	49.0	17
Anterior body end to dorsal kinety 2, distance	8.0	5.4	6.0	2.6	0.6	47.6	2.0	10.0	17
Dorsal kinety 2, number of bristles	52.0	47.1	45.0	4.4	1.1	9.3	42.0	54.0	17
Anterior body end to dorsal kinety 3, distance	5.0	4.1	3.0	2.7	0.7	66.6	1.0	10.0	17
Dorsal kinety 3, number of bristles	48.0	42.1	41.0	4.7	1.1	11.1	37.0	49.0	17
Dorsal bristles, total number	141	131.1	126.0	9.2	2.2	7.0	120.0	146.0	17
Caudal cirri, number	3	3.1	3.0	0.3	0.1	9.7	3.0	4.0	21

^a^ Data based, if not stated otherwise, on mounted, protargol-impregnated, and randomly selected specimens from a semipure culture. Measurements in µm. Abbreviations: AZM, adoral zone of membranelles; BC, buccal cirrus; CV, coefficient of variation in %; EM, endoral membrane; HT, holotype; M, median; Max, maximum; Mean, arithmetic mean; Min, minimum; *n*, number of individuals investigated; PM, paroral membrane; SD, standard deviation; SE, standard error of arithmetic mean.

Invariably two frontal cirri, with cilia ~ 14 µm long in vivo: frontal cirrus 1 (originating from anlage I) is located posterior of frontal cirrus 2 (originating from anlage II). Usually one buccal cirrus (2 of 19 specimens with two buccal cirri each), located ~ 1 µm posterior to the left of the anterior end of endoral membrane. Parabuccal row short with 6–12 cirri, commencing anteriorly at ~ 10% of body length and extending along the narrowed portion of body. Left frontoventral row with 57–74 cirri, commencing at ~ 20% of body length and ending terminally; right frontoventral row with 55–75 cirri, commencing at ~ 14% of body length and ending terminally. Both rows spiraling approximately half to one turn around body axis. Left marginal row extending from posterior portion of adoral zone to posterior body end, composed of 46–57 cirri; right marginal row commencing anteriorly at ~ 36% of body length, composed of 43–59 cirri. Both marginal rows curve approx. half a turn around long axis, ending terminally (Figs 1A, 1C, 1D, 2D–H, 3A–I, 4A, 4B; Table 1).

Three dorsal kineties, bristles 4.0–5.0 µm long in vivo. Kinety 1 slightly shortened anteriorly (i.e., commencing at ~ 13% of body length), composed of 38–49 dikinetids; kineties 2 and 3 bipolar, with 42–54 and 37–49 dikinetids, respectively. Often 2–4 bristles were observed between kineties 2 and 3; however, it is unclear whether these are anteriormost bristles of kinety 3, which bends backwards, or are parental remnants. Three, rarely four, caudal cirri, one at the posterior end of each dorsal kinety (Figs 1C, 1D, 3B–F, 4A, 4B; Table 1).

### Divisional morphogenesis

**Stomatogenesis and development of frontoventral cirral rows**

**Figure 5.**
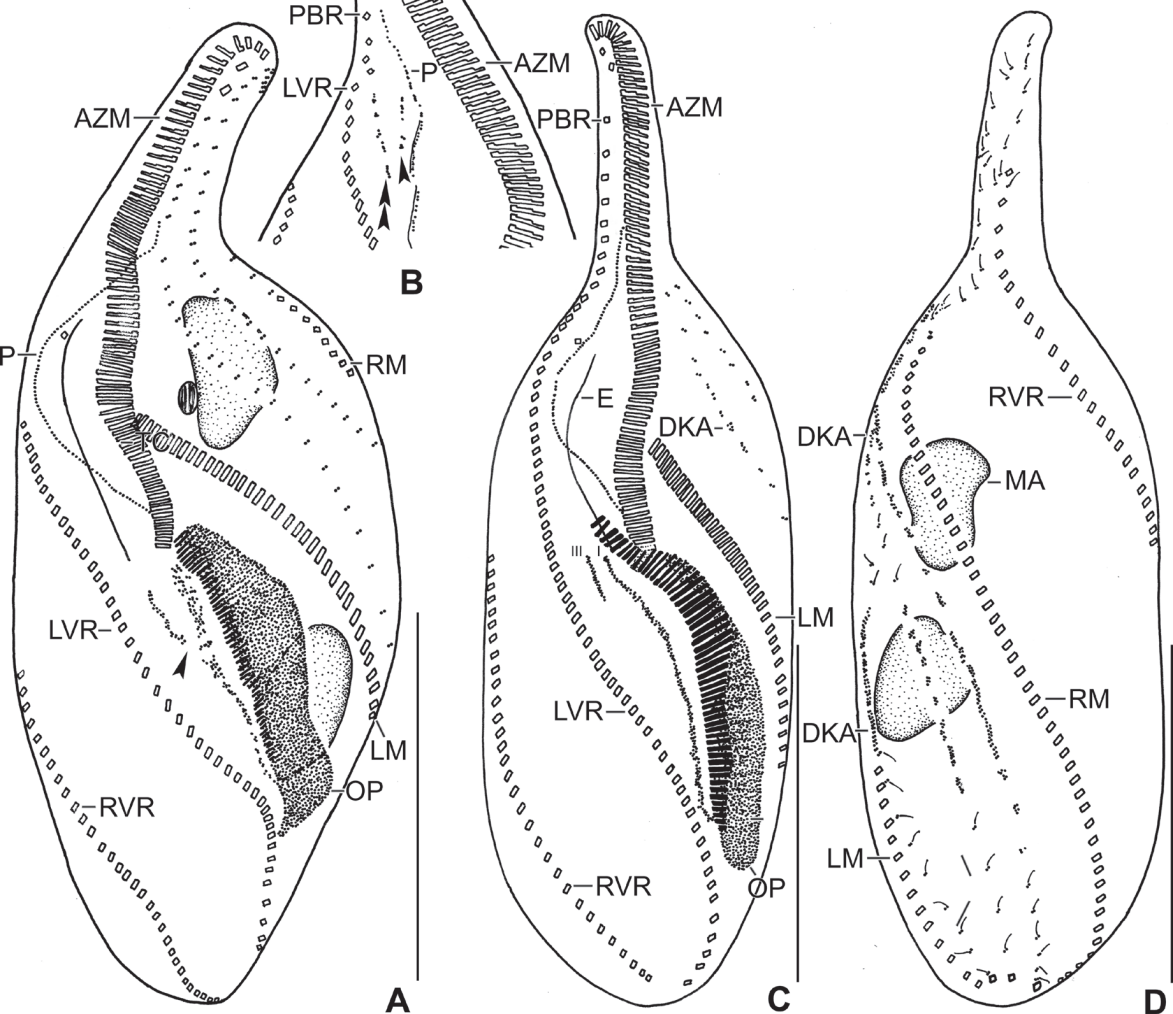
Line drawings of protargol-impregnated dividers of *Stichotricha koreana* sp. nov. **A**. Ventral view of an early divider, showing morphogenetic events on ventral surface. Note proliferation of oral primordium and formation of two anlagen, with right segment forming opisthe anlage III (arrowhead); **B**. Anlage II (arrowhead) of the proter is formed by the disaggregation of the parental buccal cirrus, while anlage III (double arrowheads) develops through the disaggregation of the posterior two or three cirri of the parabuccal row; **C, D**. Ventral (**C**) and dorsal (**D**) view of an early divider, showing the new adoral membranelles differentiating posteriorly near to the end of the oral primordium and the within-row anlagen begins to form at two levels in each dorsal kinety. Abbreviations: AZM, adoral zone of membranelles; DKA, dorsal kinety anlage; E, endoral membrane; LVR, left frontoventral row; LM, left marginal row; MA, macronuclear nodules; OP, oral primordium; P, paroral membrane; PBR, parabuccal cirral row; RM, right marginal row; RVR, right frontoventral row. Scale bars: 50 µm.

**Figure 6.**
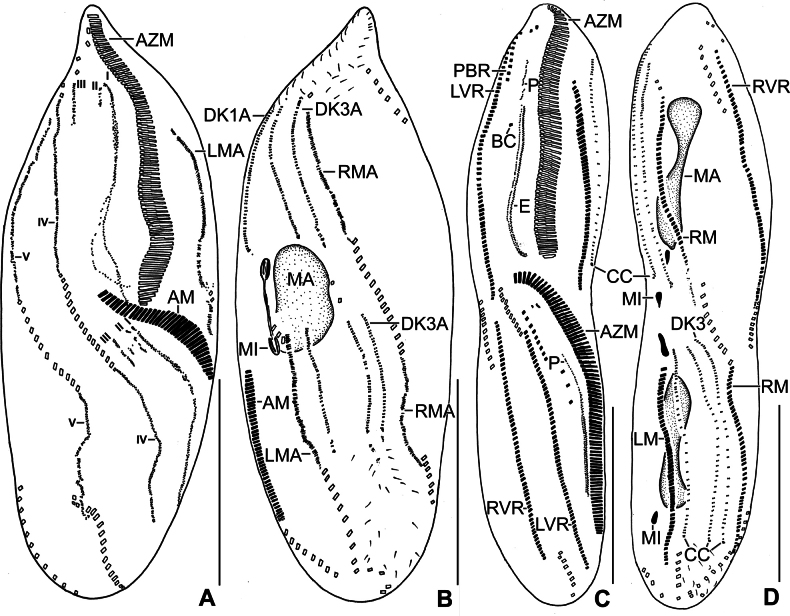
Line drawings of protargol-impregnated middle (A, B) and late (C, D) dividers of *Stichotricha koreana* sp. nov. **A, B**. Ventral (**A**) and dorsal (**B**) view showing the formation of anlagen I–III for proter and opisthe. Opisthe anlagen I–III generated from the oral primordium. Note anlagen for frontoventral rows, marginal rows, and dorsal kineties originate within-row at two levels. Macronuclear nodules fuse into a single mass; **C, D**. Anlage I produces undulating membranes and frontal cirrus 1; anlage II produces frontal cirrus 2 and buccal cirri; anlage III forms short parabuccal row, but no frontal cirrus. Three caudal cirri, each formed at the posterior end of a newly formed dorsal kinety. At this stage, macronuclear nodules begin second division, and micronuclei divide mitotically. Abbreviations: AM, adoral membranelles, AZM, adoral zone of membranelles; BC, buccal cirrus; CC, caudal cirri; DK3, dorsal kinety 3; DK1A, 3A, dorsal kineties 1, 3 anlagen; E, endoral membrane; LM, left marginal row; LMA, left marginal row anlage; LVR, left frontoventral row; MA, macronuclear nodules; MI, micronuclei; P, paroral membrane; PBR, parabuccal cirral row; RM, right marginal row; RMA, right marginal row anlage; RVR, right frontoventral row. Roman numerals denote cirral anlagen. Scale bars: 50 µm.

**Figure 7.**
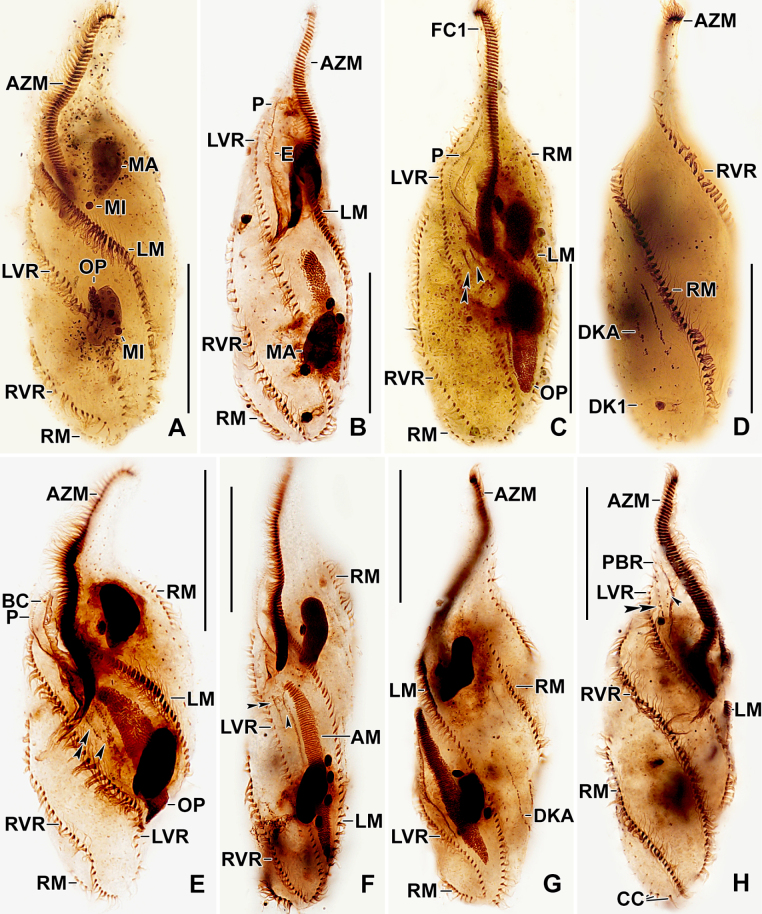
Photomicrographs of protargol-impregnated, early dividers of *Stichotricha koreana* sp. nov. **A, B**. Ventral views showing the formation of oral primordium (**A**) and the disoriented basal bodies right to anterior portion of oral primordium to form anlagen I–III; **C–G**. Proliferation of oral primordium and formation of three anlagen each for proter and opisthe. Arrowhead in C, E, and F indicates the anlage II, which originates from anlage I, while double arrowhead indicates anlage III. Note the within-row formation of ventral, marginal, and dorsal kinety rows; **H**. Arrowhead points to the formation of anlage II for the proter via disaggregation of buccal cirrus; double arrowheads indicate formation of anlage III for the proter through disaggregation of the 3–5 posterior cirri of parabuccal row. Abbreviations: AM, adoral membranelles; AZM, adoral zone of membranelles; BC, buccal cirrus; CC, caudal cirri; DKA, dorsal kinety anlage; E, endoral membrane; FC1, frontal cirrus 1; LVR, left frontoventral row; LM, left marginal row; MA, macronuclear nodules; MI, micronuclei; OP, oral primordium; P, paroral membrane; PBR, parabuccal cirral row; RM, right marginal row; RVR, right frontoventral row. Scale bars: 50 µm.

**Figure 8.**
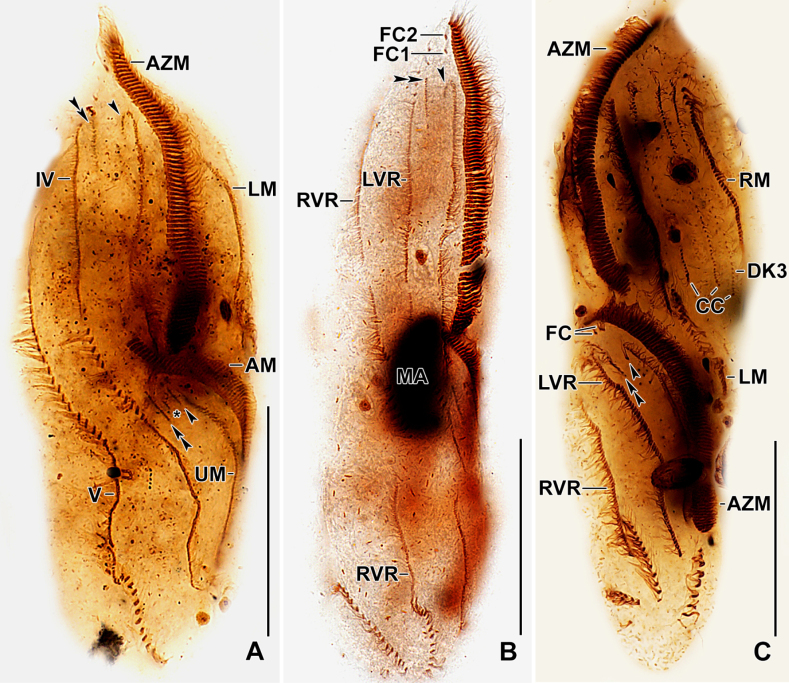
Photomicrographs of protargol-impregnated dividers of *Stichotricha koreana* sp. nov. **A–C**. Middle (**A, B**) and late (**C**) divider showing the formation of three anlagen for the proter (arrowhead points to anlage II; double arrowheads to anlage III) and three anlagen for opisthe, originating from oral primordium. Asterisk in (**A**) indicates a short additional anlage observed on several occasions in the opisthe, which later joins with the proliferating anlage II. Anlagen for frontoventral rows; marginal rows; and dorsal kineties are formed within-row at two levels. Macronuclear nodules fuse into a single mass (**A, B**) and then divide two times to form two nodules in each daughter cell in later stage (**C**). Abbreviations: AM, adoral membranelles; AZM, Adoral zone of membranelles; CC, caudal cirri; DK3, dorsal kinety 3; FC1, 2, frontal cirri; LVR, left frontoventral row; LM, left marginal row; MA, macronuclear nodules; RM, right marginal row; RVR, right frontoventral row; UM, undulating membranes. Roman numerals denote cirral anlagen. Scale bars: 50 µm.

**Figure 9.**
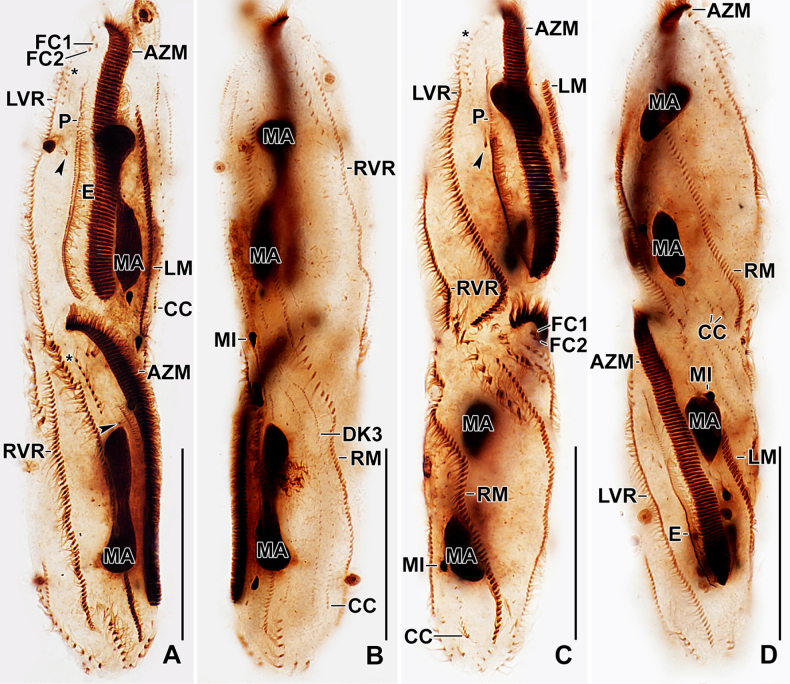
Photomicrographs of protargol-impregnated, late dividers of *Stichotricha koreana* sp. nov. **A–D**. Specimens showing events on ventral (**A, C**) and dorsal (**B, D**) surfaces. Arrowheads in (**A, C**) point to newly formed buccal cirri; asterisk in (**A**) indicates newly-formed short parabuccal row. Note that anlage I produces undulating membranes and frontal cirrus 1, anlage II produces frontal cirrus 2 and buccal cirrus, and anlage III forms parabuccal row, but no frontal cirrus. Three caudal cirri, each is formed at posterior end of a newly developed dorsal kinety. At this stage, macronuclear nodules complete their second division, resulting in two nodules per daughter cell. Abbreviations: AZM, adoral zone of membranelles; CC, caudal cirri; DK3, dorsal kinety 3; E, endoral membrane; FC1, 2, frontal cirri; LVR, left frontoventral row; LM, left marginal row; MA, macronuclear nodules; MI, micronuclei; P, paroral membrane; RM, right marginal row; RVR, right frontoventral row. Scale bars: 50 µm.

Cell division begins with the proliferation of the oral primordium behind the parental adoral zone of membranelles, immediately left of 4–7 cirri in the posterior third of left frontoventral row (Figs 4C, 4D, 7A, 7B). Subsequently, the oral primordium enlarges; a disoriented row of basal bodies splits from its right side to form anlagen I–III, and the new adoral membranelles start to develop in the right anterior portion, extending posteriorly (Figs 4D, 7B, 7C). The disoriented row right of the oral primordium splits anteriorly to form anlagen I and III (Figs 5A, 7B, 7C). In mid dividers, the parental undulating membranes disaggregate to form anlage I of the proter. Anlage II of the proter originates from the disaggregation of the parental buccal cirri and migrates anteriorly to the right of the anterior portion of anlage I (Figs 5B, 7H). Anlage III of the proter develops by disaggregation of the posterior two or three cirri of the parabuccal row (Figs 5B, 7H). A longitudinal streak of basal bodies forms right of, and likely from, the anterior portion of anlage I of the opisthe forming anlage II (Figs 5A, 5C, 6A, 7C, 7E, 7F, 8A, 8B). Simultaneously, anlagen IV and V for both daughter cells are formed within-row from the left and right frontoventral rows at two levels, respectively (Figs 6A, 6B, 8A–C, 9A–D). Later, anlage I in each daughter cell produces the frontal cirrus 1, and the paroral and endoral membranes. Anlage II produces the frontal cirrus 2 and buccal cirri in both proter and opisthe, then frontal cirrus 2 migrates anteriorly in front of frontal cirrus 1. Anlagen III of the proter and opisthe form the parabuccal row in each daughter cell. The parental adoral zone is retained for the proter, while the adoral zone of membranelles of the opisthe develops from the oral primordium (Figs 6C, 6D, 8C, 9A, 9C).

**Development of marginal cirral rows**

Marginal primordia form at two levels by ‘within-row’ anlagen formation, each originating from two to four parental cirri. These primordia elongate by incorporating six to twelve parental cirri each and develop into new marginal rows, while the remaining parental cirri are resorbed (Figs 6A–D, 8A–C, 9A–D).

**Development of dorsal ciliature**

On the dorsal surface, three anlagen develop ‘within-row’ from dorsal kineties 1–3 at two levels, forming one set for each daughter cell (proter and opisthe). A single caudal cirrus arises at the posterior end of each new kinety (Figs 5D, 6B, 6D, 7D, 7G, 8C, 9B).

**Division of nuclear apparatus**

Nuclear division follows the typical hypotrich pattern. The macronuclear nodules fuse into a single mass during mid-division, which then divide twice to form the typical four nodules in the late stages. The micronuclei undergo standard mitotic division (Figs 6B, 6D, 8B, 8C, 9A–D).

### Phylogenetic analyses

**Figure 10.**
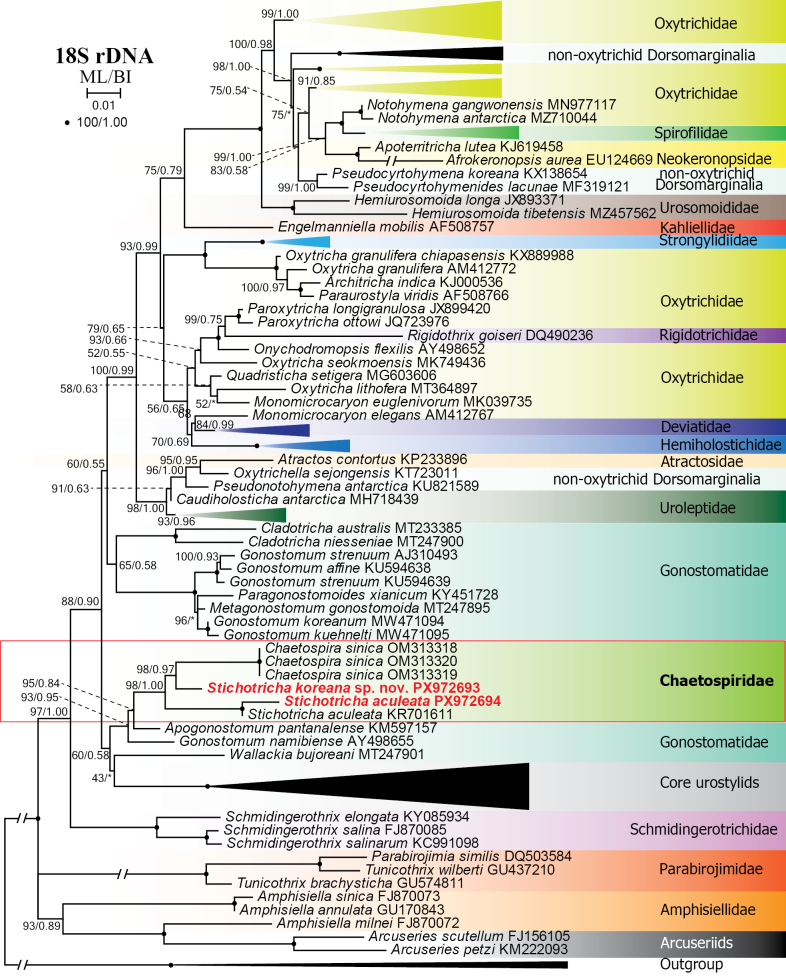
Maximum likelihood tree based on 18S rDNA sequences showing the phylogenetic position of *Stichotricha koreana* sp. nov. and *S. aculeata*. Newly obtained sequences are in bold red. GenBank accession numbers are given after species names. Numbers at nodes represent the maximum likelihood bootstrap values and the Bayesian inference posterior probabilities. Asterisks (*) indicate different topologies between BI and ML analyses. Accession numbers of the taxa in the compressed clades are listed in the Suppl. material 1. The scale bar represents one substitution per 100 nucleotide positions.

**Figure 11.**
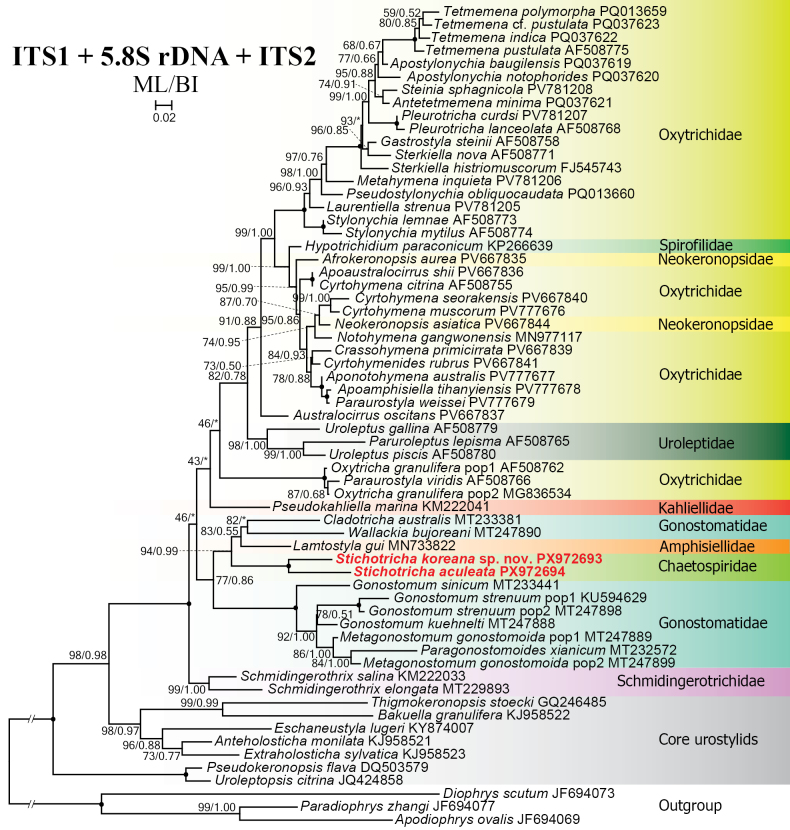
Maximum likelihood tree based on ITS1–5.8S rDNA–ITS2 sequences showing the phylogenetic position of *Stichotricha koreana* sp. nov. and *S. aculeata*. Newly obtained sequences are in bold red. GenBank accession numbers are given after species names. Numbers at nodes represent the maximum likelihood bootstrap values and the Bayesian inference posterior probabilities. Asterisks (*) indicate different topologies between BI and ML analyses. The scale bar represents two substitutions per 100 nucleotide positions.

**Figure 12.**
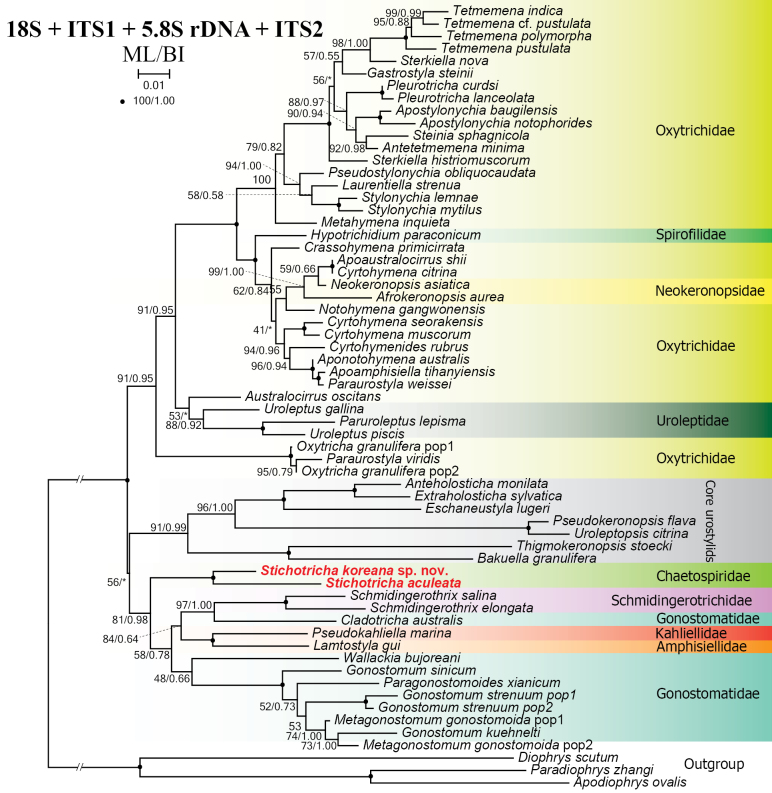
Maximum likelihood tree based on 18S–ITS1–5.8S–ITS2 rDNA sequences showing the phylogenetic position of *Stichotricha koreana* sp. nov. and *S. aculeata*. Newly obtained sequences are in bold red. Numbers at nodes represent the maximum likelihood bootstrap values and the Bayesian inference posterior probabilities. Asterisks (*) indicate different topologies between BI and ML analyses. The scale bar represents one substitution per 100 nucleotide positions.

**Figure 13.**
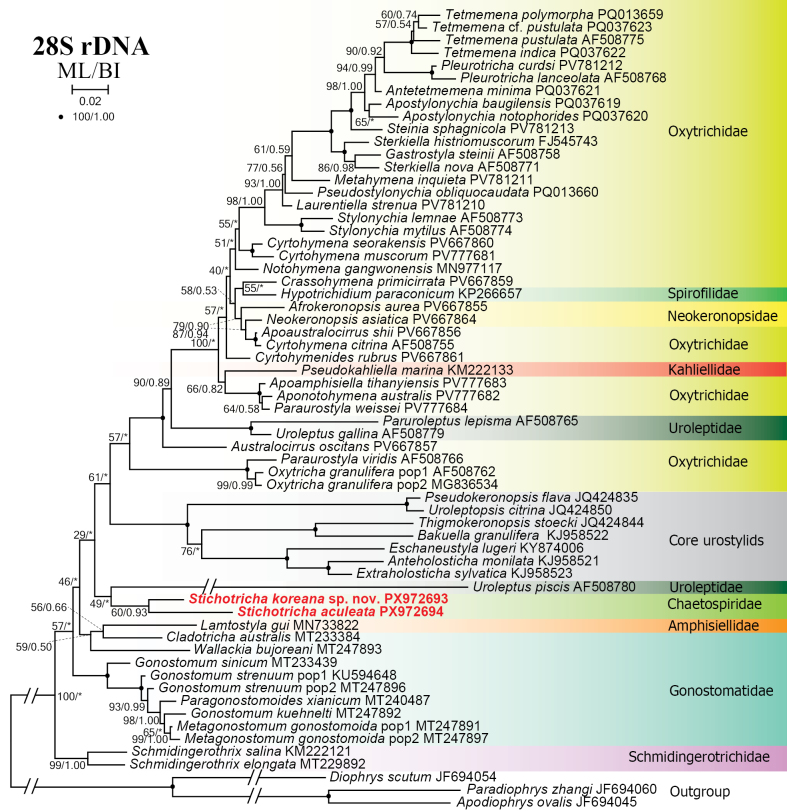
Maximum likelihood tree based on 28S rDNA sequences showing the phylogenetic position of *Stichotricha koreana* sp. nov. and *S. aculeata*. Newly obtained sequences are in bold red. GenBank accession numbers are given after species names. Numbers at nodes represent the maximum likelihood bootstrap values and the Bayesian inference posterior probabilities. Asterisks (*) indicate different topologies between BI and ML analyses. The scale bar represents two substitutions per 100 nucleotide positions.

**Figure 14.**
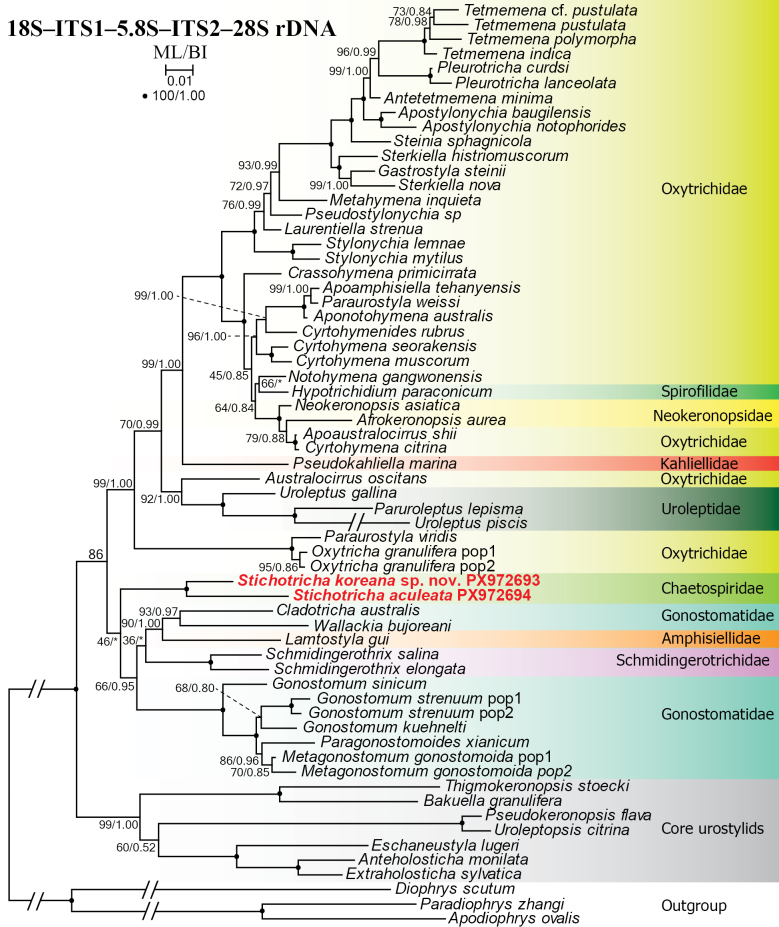
Maximum likelihood tree inferred from the concatenated 18S–ITS1–5.8S–ITS2–28S rDNA sequences showing the phylogenetic position of *Stichotricha koreana* sp. nov. and *S. aculeata*. Newly obtained sequences are in bold red. GenBank accession numbers are given after species names. Numbers at nodes represent the maximum likelihood bootstrap values and the Bayesian inference posterior probabilities. Asterisks (*) indicate different topologies between BI and ML analyses. The scale bar represents one substitution per 100 nucleotide positions.

The 18S–ITS1–5.8S–ITS2–28S rDNA sequences obtained from *Stichotricha koreana* sp. nov. and *S. aculeata* have been deposited in the GenBank database with accession numbers PX972693 and PX972694, respectively. The two sequences are 5336 and 5311 bp long and with GC content of 47.54% and 46.75%, respectively, and show a similarity of 96.51% (185 nucleotide differences). The trees constructed using maximum likelihood (ML) and Bayesian inference (BI) analyses show very similar topologies except that of the 28S rDNA, which shows highly variable topologies. However, only the ML tree is presented, with bootstrap values (ML) and posterior probabilities (BI) indicated (Figs 10, 11, 12, 13, 14). In the 18S rDNA tree, the sequence of *S. koreana* sp. nov. shows a sister relationship to the clade of three identical sequences of *Chaetospira sinica* Song et al., 2022 with high (98% ML, 0.97 BI) supporting values, and show similarity of 98.41% (25 nucleotide differences). The 18S rDNA sequence of the Korean population of *S. aculeata* clusters with that of the North American population of the same species with full support. The two sequences of *S. aculeata* show a similarity of 99.43% (9 nucleotide differences). The 18S rDNA sequence of *S. koreana* sp. nov. shows similarity of 98% and 99.4% (30 and 23 nucleotide differences) with the Korean and American populations of *S. aculeata*, respectively. They cluster together with very high (98% ML, 1.00 BI) supporting values. The chaetospirid clade clusters with two gonostomatid sequences, *Apogonostomum pantanalense* Foissner, 2016 (KM597157) and *Gonostomum namibiense* Foissner et al., 2002 (AY498655) with moderate to low support (95% ML, 0.84 BI and 93% ML, 0.95 BI, respectively). The chaetospirids and the two gonostomatid sequences cluster with a clade containing *Wallackia bujoreani* (Lepsi, 1951) Berger & Foissner, 1989 (MT247901) and the core urostylids with very low (60% ML, 0.58 BI) supporting values. The core gonostomatids form a sister clade to the dorsomarginalian assemblage (Fig. 10).

In the phylogenetic tree constructed using the ITS1–5.8S–ITS2 region, the two new sequences of *Stichotricha* cluster together with full support and nest as a sister to *Lamtostyla gui* Liao et al., 2020 (MN733822), *Cladotricha australis* Blatterer & Foissner, 1988 (MT233381), and *Wallackia bujoreani* (MT247890) with medium (94% ML) to high (0.99 BI) support. Together, these five sequences form a sister clade to the clade containing the core gonostomatids with medium (77% ML) to low (0.86 BI) support (Fig. 11). In the phylogenetic tree constructed using both the 18S rDNA and ITS1–5.8S–ITS2 region, the sequences of *S. koreana* sp. nov. and *S. aculeata* nest at the base of a clade containing the core gonostomatid clade, which also host sequences of *Pseudokahliella marina* (Foissner et al., 1982) Berger et al., 1985 and *Lamtostyla gui*, with medium (81% ML) to high (0.98 BI) support (Fig. 12). In the phylogenetic tree constructed using the 28S rDNA sequences, the position of the chaetospirid species is incongruent in ML and BI analyses. In the ML tree, *Stichotricha koreana* sp. nov. and *S. aculeata* cluster with *Uroleptus piscis* (Müller, 1773) Ehrenberg, 1831, which deviates from its usual position among congeners in other datasets, without support, whereas in the BI tree, *Stichotricha* spp. nest in a polytomy with urostylids, gonostomatids, and the dorsomarginalian assemblage (Fig. 13).

In the phylogenetic tree constructed using concatenated 18S–ITS1–5.8S–ITS2–28S rDNA sequences, *Stichotricha koreana* sp. nov. clusters with *S. aculeata* with full support. Together, the two sequences form a sister clade to the gonostomatid clade without support in the ML analyses and form a polytomy with the gonostomatids and the Dorsomarginalia in the BI analyses (Fig. 14).

## Discussion

### Notes on the generic assignment of *Stichotricha koreana* sp. nov.

A spiraled body is shared among several hypotrich families such as the Atractosidae Bourland, 2015, Chaetospiridae, Schmidingerotrichidae Foissner, 2012, Spirofilidae Gelei, 1929, and Strongylidiidae Fauré-Fremiet, 1961. Each of these families is diagnosed by distinct morphological and morphogenetic traits. Atractosidae is characterized by a morphogenetically inactive right marginal row that derives from the right ventral row anlage (Bourland 2015). Spirofilidae shows a de novo origin of most ventral cirral anlagen and all dorsal anlagen, together with dorsal kinety fragmentation (Chen et al. 2013; Luo et al. 2022; Omar et al. 2025). Schmidingerotrichidae lacks both dorsal bristles and paroral membrane (Foissner 2012; Foissner et al. 2014, 2018; Lu et al. 2018). Strongylidiidae is marked by the mixed origin of the ventral cirral rows from different anlagen (Tuffrau and Fleury 1994; Shi 1999; Lynn and Small 2002; Jankowski 2007; Lynn 2008; Luo et al. 2018).

In their improved diagnosis of Chaetospiridae, Song et al. (2022) emphasized features that overlap with other spiraled-bodied hypotrichs (e.g., number of cirral rows; absence of pretransverse and transverse cirri). A decisive family-level discriminator that was underemphasized, is the gonostomatid oral apparatus: an adoral zone running along the left body margin together with a stichomonad paroral membrane composed of widely spaced kinetosomes and a stichomonad endoral composed of densely spaced kinetosomes (Berger 2011; Foissner 2016; Omar et al. 2021). On the basis of this oral pattern, together with its morphogenetic characters, *Stichotricha koreana* sp. nov. fits the Chaetospiridae (see revised family diagnosis above). Within the family Chaetospiridae, the new species, *Stichotricha koreana* sp. nov. can be easily differentiated from members of the genus *Chaetospira* by the presence (vs absence) of frontal and buccal cirri and the straight (vs cork-screw shaped) adoral zone of membranelles.

The few *Stichotricha* species with adequate morphological data segregate into two groups. (i) Group I consists of *S. koreana* sp. nov. and *S. marina*, characterized by having a parabuccal cirral row, two long frontoventral cirral rows, and three dorsal kineties, each terminating in a caudal cirrus (Hu and Song 2001). (ii) Group II consists of *S. aculeata*, *S. intermedia*, and *S. multinucleata*, lacks both the parabuccal cirral row and the caudal cirri (Wang and Nie 1933; Fernandez-Leborans 1985; Song and Wilbert 1989; Hu and Song 2001; Bourland 2015). These stable differences suggest that the two groups likely represent distinct genera. This interpretation is further supported by our molecular results: *S. koreana* sp. nov. clusters with *Chaetospira sinica* rather than with its nominal congener *Stichotricha aculeata*, implying that the morphological gap between *S. koreana* sp. nov. and *C. sinica* is smaller than, or comparable to, that between *S. koreana* sp. nov. and *S. aculeata* (Song et al. 2022). Formal generic revision, however, should await a modern redescription and sequencing of the type species, *Stichotricha secunda*, which remains insufficiently characterized. Without robust morphological and molecular data for *S. secunda*, dividing *Stichotricha* would be premature.

### Comparison of *Stichotricha koreana* sp. nov. with congeners

Most species in the genus *Stichotricha* require redescriptions using modern techniques for improved comparison with similar species. Currently, only *Stichotricha aculeata* has been thoroughly described with molecular sequences (Bourland 2015; Omar and Jung 2021). The remaining species lack detailed descriptions of their primary characteristics, complicating species identification (see Berger 2001 for details). Based on the available data and comparison of key morphometric features with species of *Stichotricha*, the present species has been identified as new to science.

The type species of the genus, *Stichotricha secunda*, can be distinguished from the new species and other congeners by having endosymbiotic zoochlorellae and long dorsal bristles, i.e., 20 µm (Perty 1852; Kahl 1932; Froud 1949; Foissner et al. 1991). *Stichotricha koreana* sp. nov. can be differentiated from *S. aculeata* by having three (vs 2) dorsal kinety rows, and in the presence (vs absence) of parabuccal cirral row and caudal cirri. However, it should be noted that the two species agree in having two frontal cirri because two of the four cirri recognized by Foissner et al. (1991) and Bourland (2015) are in line with the left frontoventral cirral row. Despite the high morphological similarity between the Korean and American populations of *S. aculeata*, their 18S rDNA sequences differ by nine nucleotides (Bourland 2015; Omar and Jung 2021). This suggests a possible synonymy, but detailed description and ontogenetic studies are required to resolve this issue and clarify the diversity within *S. aculeata*-like complex. *Stichotricha marina* is the most similar congener to *S. koreana* sp. nov. However, they can be distinguished by the contractility of the body (absent vs present); the bending of the anterior end to right (absent vs present); in having more cirri in parabuccal row (~35 including the buccal cirrus vs ~10), however, Hu and Song (2001) considered this row as a row of buccal cirri; single (vs branched) lorica; and habitat (marine vs terrestrial) (Hu and Song 2001).

*Stichotricha asensius* is distinguishable from *S. koreana* sp. nov. by the absence (vs presence) of frontal cirri and by having a paroral membrane segmented into two parts (vs continuous) (Fernandez-Leborans 1985). *Stichotricha gracilis* differs from *S. koreana* sp. nov. in several aspects: it has a smaller body length in vivo (100 µm vs 194 µm), the absence (vs presence) of lorica, and habitat (marine vs semi-terrestrial) (Möbius 1888). *Stichotricha saginata* mainly differs from *S. koreana* in having four (vs 2) macronuclear nodules and the habitat (marine vs semi-terrestrial) (Möbius 1888). *Stichotricha koreana* sp. nov. differs from *S. intermedia* by the presence (vs absence) of frontal cirri, three (vs two) dorsal kineties, and the presence (vs absence) of a lorica (Froud 1949). *Stichotricha multinucleata* can be separated from *Stichotricha koreana* sp. nov. by the higher number of macronuclear nodules (9–15 vs 2), the lower number of adoral membranelles (22–28 vs 71–94), and dorsal kineties (2 vs 3), and the absence (vs presence) of a parabuccal cirral row and caudal cirri (Song and Wilbert 1989). According to the drawings of Wang and Nie (1933), the poorly known *Stichotricha nankingensis* possesses a short adoral zone of membranelles forming a question mark and its undulating membranes are strongly curved leftward and slightly recurved distally as in the *Cyrtohymena* pattern. These characters suggest that *S. nankingensis* does not belong to the family Chaetospiridae, in which all members exhibit gonostomatid oral apparatus (Wang and Nie 1933). *Stichotricha opisthotonoides* was synonymized with *S. aculeata* by Borror (1972), however, its body shape, the length of the adoral zone relative to body length, and the location of the contractile vacuole suggest that it is a distinct species as suggested by Kahl (1932). It can be differentiated from *S. koreana* sp. nov. in the body shape (club-shaped vs slender fusiform), the length of the adoral zone of membranelles (~70% of body length vs 58%), and the location of contractile vacuole (near rear end vs near mid-body) (Smith 1897; Kahl 1932; Berger 2001;) *Stichotricha tubicola* can be differentiated from *S. koreana* sp. nov. by the smaller body size in vivo (55–97 vs 172–217), the fewer adoral membranelles (~ 40 vs 79) and cirri in right (~ 30 vs 53) and left (~ 20 vs 52) marginal rows (Dingfelder 1962).

### The phylogenetic position of the family Chaetospiridae

Earlier phylogenetic studies placed Chaetospiridae as sister to urostylids when gonostomatids were sparsely sampled or absent (Song et al. 2022; Hong et al. 2025), and as sister to the Dorsomarginalia together with Gonostomatidae when urostylid sequences were not included in the analyses (Bourland 2015). Expanded taxon sampling, both within Chaetospiridae and among taxa bearing a gonostomatid-like oral apparatus (Gonostomatidae, Schmidingerotrichidae), highlights a close affinity among these families. Nevertheless, in our 18S rDNA tree, Chaetospiridae clade clusters only with *Apogonostomum pantanalense* and *Gonostomum namibiense*, whereas *Wallackia bujoreani* falls closer to the core urostylids, albeit with low support (Fig. 10). Notably, *A. pantanalense* and *G. namibiense* are tailed gonostomatids that frequently group closer to the urostylids than to the core gonostomatid clade and differ markedly from chaetospirids by possessing pretransverse and transverse cirri and midventral cirral pairs (Ning et al. 2019; Park et al. 2020; Wang et al. 2020; Omar et al. 2021). Resolving these placements will require denser taxon sampling and morphogenetic data for tailed gonostomatids.

Species of *Wallackia* Foissner, 1976 are morphologically and morphogenetically very similar to chaetospirids: they share a gonostomatid oral apparatus; two frontoventral rows; a parabuccal cirral row (originating from the oral primordium in the opisthe and within-row in the proter as in *Stichotricha koreana* sp. nov.); one marginal row on each side; and three dorsal kineties. Frontoventral, marginal, and dorsal rows formed at two levels via within-row anlagen. The main distinctions from Chaetospiridae include an ordinary (non-spiraled) body (vs spiraled), a continuous buccal cirral row (vs a few buccal cirri), and the absence (vs presence) of loricae. Moreover, Berger (2011) argued that the “transverse cirri” sensu stricto are lacking in the genus *Wallackia* and the posteriormost cirri of the frontoventral rows having been misinterpreted (see also Foissner et al. 2002).

Across alternative datasets, where tailed gonostomatids are not represented, the new sequences of *Stichotricha koreana* sp. nov. and *S. aculeata* are placed: (i) as sister to the Gonostomatidae + Schmidingerotrichidae clade (18S–ITS1–5.8S–ITS2 rDNA and concatenated rDNA trees; Figs 12, 14, respectively); (ii) within the core Gonostomatidae clade (ITS1–5.8S–ITS2 tree; Fig. 11); or (iii) outside, distant from core Gonostomatidae (28S rDNA tree; Fig. 13). The extremely long branch and aberrant position of *Uroleptus piscis* in the 28S rDNA tree are most likely due to sequencing problems, and may artificially contribute to the separation of Chaetospiridae from both gonostomatids and urostylids in this marker. Taken together, these topologies support a close relationship between the Chaetospiridae and Gonostomatidae, while also indicating residual instability among shallow nodes. *Wallackia* typically emerges as sister to core Gonostomatidae, often near the genus *Cladotricha*. The proximity of *Wallackia* and *Cladotricha* in most phylogenetic analyses is consistent with their high morphological similarity. However, their relationship with the core Gonostomatidae and among each other requires further taxon sampling and more morphological and ontogenetic studies.

## Supplementary Material

XML Treatment for Chaetospiridae

XML Treatment for Stichotricha koreana

## Additional information

### Use of AI

The authors used ChatGPT (OpenAI) solely to enhance the clarity and language of the manuscript. All content was subsequently reviewed and edited by the authors, who take full responsibility for the final version of the paper.

### Author ORCIDs

Daizy Bharti https://orcid.org/0000-0002-8311-4657

Santosh Kumar https://orcid.org/0000-0003-0378-400X

Atef Omar https://orcid.org/0000-0002-7759-632X

Jae-Ho Jung https://orcid.org/0000-0001-5497-8678

### Data availability

All of the data that support the findings of this study are available in the main text or Supplementary Information.

## Supplementary materials

Supplementary material 1 Taxon names and GenBank accession numbers used in the phylogenetic analyses not presented in Fig. 10. Data type docx https://binary.pensoft.net/file/1579037This dataset is made available under the Open Database License (http://opendatacommons.org/licenses/odbl/1.0/). The Open Database License (ODbL) is a license agreement intended to allow users to freely share, modify, and use this Dataset while maintaining this same freedom for others, provided that the original source and author(s) are credited. Daizy Bharti, Santosh Kumar, Atef Omar, Jae-Ho Jung
