# Supplementary material for: Morphology, morphogenesis, and multigene phylogeny of Stichotricha koreana sp. nov. (Alveolata, Ciliophora, Hypotricha): taxonomic implications for the family Chaetospiridae Jankowski in Small & Lynn, 1985
Source: Zookeys. 2026 Apr 2;1275:345–73. doi: 10.3897/zookeys.1275.181694 (PMC13066933; doi:10.3897/zookeys.1275.181694)
Supplement: Supplementary material 1 — Taxon names and GenBank accession numbers used in the phylogenetic analyses not presented in Fig. 10. [file zookeys-1275-345_article-181694__-s001.docx]

**Supplementary Table S1.** Taxon names and GenBank accession numbers used in the phylogenetic analyses not presented in Fig. 10.

| Taxon name | GenBank accession number |
| --- | --- |
| *Anteholosticha antecirrata* | KU234525 |
| *Anteholosticha manca* | DQ503578 |
| *Anteholosticha monilata* | KJ958488 |
| *Anteholosticha paramanca* | KF806443 |
| *Apoamphisiella vernalis* | KU522216 |
| *Apobakuella fusca* | JN008942 |
| *Apodiophrys ovalis* | GU477634 |
| *Apokeronopsis wrighti* | EU417963 |
| *Aponotohymena apoaustralis* | KC430934 |
| *Aponotohymena australis* | MG994983 |
| *Aponotohymena isoaustralis* | KP336402 |
| *Cyrtohymenides aspoecki* | MN562621 |
| *Cyrtohymenides australis* | KP100452 |
| *Cyrtohymenides australis* | MN559078 |
| *Australothrix xianiensis* | KT892731 |
| *Bakuella granulifera* | KJ958489 |
| *Bakuella litoralis* | KR024010 |
| *Bakuella subtropica* | KC631826 |
| *Birojimia soyaensis* | MK959231 |
| *Caudiholosticha stueberi* | KT724201 |
| *Cyrtohymena citrina* | KC182574 |
| *Cyrtohymena muscorum* | KM061384 |
| *Deviata bacilliformis* | KJ766110 |
| *Deviata brasiliensis* | KP266620 |
| *Diaxonella pseudorubra* | GU942564 |
| *Diaxonella trimarginata* | JQ424833 |
| *Diophrys scutum* | JF694040 |
| *Gastrostyla steinii* | AF508758 |
| *Hemiholosticha kahli* | MK211833 |
| *Histriculus histrio* | FM209294 |
| *Hypotrichidium conicum* | MW830115 |
| *Hypotrichidium paraconicum* | JQ918371 |
| *Hypotrichidium tisiae* | ON117316 |
| *Laurentiella strenua* | AJ310487 |
| *Metahymena inquieta* | KM923764 |
| *Metasterkiella koreana* | KY448243 |
| *Metastylonychia nodulinucleata* | KY353799 |
| *Metaurostylopsis alrasheidi* | MT911525 |
| *Metaurostylopsis cheni* | FJ775720 |
| *Neourostylopsis flava* | KR013238 |
| *Nothoholosticha flava* | KR612271 |
| *Onychodromopsis flexilis* | AM412764 |
| *Onychodromus grandis* | AJ310486 |
| *Paradiophrys zhangi* | FJ870076 |
| *Paraparentocirrus sibillinensis* | KF184655 |
| *Parasterkiella thompsoni* | KM061387 |
| *Paraurostyla weissei* | AJ310485 |
| *Paraurostyla wuhanensis* | MN137908 |
| *Paruroleptus lepisma* | AF164132 |
| *Pattersoniella vitiphila* | JX885704 |
| *Perisincirra paucicirrata* | JX012184 |
| *Pleurotricha lanceolata* | AF164128 |
| *Polystichothrix monilata* | KT192639 |
| *Pseudogastrostyla flava* | KP266627 |
| *Pseudokeronopsis rubra* | HM140387 |
| *Pseudouroleptus caudatus* | DQ910904 |
| *Psilotrichides hawaiiensis* | MK211834 |
| *Rigidohymena candens* | KC414885 |
| *Rigidohymena quadrinucleata* | KX925220 |
| *Rubrioxytricha ferruginea* | AF370027 |
| *Rubrioxytricha guamensis* | KY947508 |
| *Rubrioxytricha haematoplasma* | KJ645977 |
| *Rubrioxytricha tsinlingensis* | KR817675 |
| *Sterkiella cavicola* | GU942565 |
| *Sterkiella histriomuscorum* | FJ545743 |
| *Sterkiella nova* | AF508771 |
| *Sterkiella sinica* | KR817676 |
| *Sterkiella subtropica* | KM924307 |
| *Strongylidium guangdongense* | MF113406 |
| *Strongylidium orientale* | KC153532 |
| *Stylonychia ammermanni* | FM209295 |
| *Stylonychia lemnae* | AF508773 |
| *Stylonychia mytilus* | AF508774 |
| *Tetmemena pustulata* | AF508775 |
| *Thigmokeronopsis stoecki* | EU220226 |
| *Uroleptus gallina* | AF164130 |
| *Uroleptus longicaudatus* | KF734979 |
| *Uroleptus piscis* | AF164131 |
| *Urospinula succisa* | KF411460 |
| *Urostyla grandis* | AF164129 |
| *Urostyla grandis* | AF508781 |
